# Comparison of the microbial composition of African fermented foods using amplicon sequencing

**DOI:** 10.1038/s41598-019-50190-4

**Published:** 2019-09-25

**Authors:** Maria Diaz, Lee Kellingray, Nwanneka Akinyemi, Oyetayo Olaoluwa Adefiranye, Arinola B. Olaonipekun, Geoffroy Romaric Bayili, Jekwu Ibezim, Adriana Salomina du Plessis, Marcel Houngbédji, Deus Kamya, Ivan Muzira Mukisa, Guesh Mulaw, Samuel Manthi Josiah, William Onyango Chienjo, Amy Atter, Evans Agbemafle, Theophilus Annan, Nina Bernice Ackah, Elna M. Buys, D. Joseph Hounhouigan, Charles Muyanja, Jesca Nakavuma, Damaris Achieng Odeny, Hagretou Sawadogo-Lingani, Anteneh Tesfaye Tefera, Wisdom Amoa-Awua, Mary Obodai, Melinda J. Mayer, Folarin A. Oguntoyinbo, Arjan Narbad

**Affiliations:** 1 Food Innovation and Health Institute Strategic Programme, Quadram Institute Bioscience, Norwich Research Park, Norwich, United Kingdom; 2 Gut Microbes and Health Institute Strategic Programme, Quadram Institute Bioscience, Norwich Research Park, Norwich, United Kingdom; 3 Department of Microbiology, Faculty of Science, University of Lagos, Lagos, Nigeria; 4 Consumer and Food Science Department, University of Pretoria, Pretoria, South Africa; 5 Département Technologie Alimentaire DTA/IRSAT/CNRST, Ouagadougou, Burkina Faso; 6 Laboratoire de Sciences des Aliments, Université d’Abomey-Calavi, Abomey-Calavi, Benin; 7 Department of Food Technology & Nutrition, Makerere University, Kampala, Uganda; 8 Department of Microbial, Cellular and Molecular Biology, Addis Ababa University, Addis Ababa, Ethiopia; 9 International Crops Research Institute for Semi-arid Tropics (ICRISAT), Nairobi, Kenya; 10 Department of Food Science and Technology, The Technical University if Kenya, Nairobi, Kenya; 11 CSIR-Food Research Institute, Accra, Ghana; 12 Institute of Biotechnology, Addis Ababa University, Addis Ababa, Ethiopia; 13 A.R. Smith Department of Chemistry and Fermentation Sciences, Appalachian State University, Boone, North Carolina USA

**Keywords:** Food microbiology, Microbial ecology

## Abstract

Fermented foods play a major role in the diet of people in Africa, where a wide variety of raw materials are fermented. Understanding the microbial populations of these products would help in the design of specific starter cultures to produce standardized and safer foods. In this study, the bacterial diversity of African fermented foods produced from several raw materials (cereals, milk, cassava, honey, palm sap, and locust beans) under different conditions (household, small commercial producers or laboratory) in 8 African countries was analysed by 16S rRNA gene amplicon sequencing during the Workshop “Analysis of the Microbiomes of Naturally Fermented Foods Training Course”. Results show that lactobacilli were less abundant in fermentations performed under laboratory conditions compared to artisanal or commercial fermentations. Excluding the samples produced under laboratory conditions, lactobacilli is one of the dominant groups in all the remaining samples. Genera within the order *Lactobacillales* dominated dairy, cereal and cassava fermentations. Genera within the order *Lactobacillales*, and genera *Zymomonas* and *Bacillus* were predominant in alcoholic beverages, whereas *Bacillus* and *Lactobacillus* were the dominant genera in the locust bean sample. The genus *Zymomonas* was reported for the first time in dairy, cereal, cassava and locust bean fermentations.

## Introduction

Traditional fermented foods play a major role in the diet of numerous communities worldwide. Africa is perhaps the continent with the richest variety of fermented foods, where fermentation still plays a major role in combating food spoilage, foodborne diseases and represents a significant postharvest value addition. In fact, fermentation is still a largely home-based process used throughout the continent^1^. A wide variety of raw materials are traditionally fermented in different regions of Africa. As a result, fermented foods with different characteristics are produced and they have been classified in groups such as fermented non-alcoholic cereals (mainly produced from sorghum, millet and maize), starchy root crops (mainly produced from cassava), animal proteins (mainly dairy products), vegetable proteins (produced from legumes and oilseeds) and alcoholic beverages (produced from cereals, sap, honey or fruits, among other materials)^1^. Fermented products have been described to provide health benefits, such as protection against gastrointestinal disorders, prevention of hypertension and heart disease or protection from diabetes and osteoporosis. In addition, traditional African fermented foods contain live microorganisms that can produce health-promoting compounds, such as antimicrobials, essential nutrients or molecules with antioxidant activity, and can act as probiotic strains^2,3^.

Knowledge about the microbial ecology of natural food fermentations can be used to identify biomarkers to assess the quality of fermented foods and would help in the design of optimum starter cultures^4^. Predominant bacterial groups present in African fermented foods have been widely analysed using culture-dependant methods^5^, but these methods present several limitations, such as not being able to detect non-culturable populations or being unable to detect microorganisms in low numbers in complex ecosystems with dominant populations^6^. As an alternative, culture-independent methods, particularly amplicon sequencing, are increasingly being used to study the bacterial populations of fermented foods, although to date, few studies have focused on African foods^7–9^.

Culture-independent methods present several biases, some of which are associated with the DNA extraction procedures used. The extraction of DNA from foods can be challenging due to the structure and chemical composition of the matrices. Therefore, due to the vast variety of raw materials that are fermented, diverse DNA extraction procedures can be found in the literature^10^. In fact, some food matrices require pre-processing steps before DNA extraction^11–13^. In order to analyse the microbial population of different fermented samples, the application of a standardized method would be beneficial. Commercial extraction kits partially solve the bias problems, but usually they have been tested for common food matrices and cannot be applied to complex foods^10^. In addition, commercial kits use small amounts of sample. This can be an advantage as less material is required, but for some types of food it may not yield enough microbial DNA, as we have experienced in previous experiments in our laboratory. Here, the use of a single method for the analysis of a wide range of African fermented products was evaluated for its performance across diverse food matrices.

Despite the important information that culture-independent methods contribute to the understanding of the complete microbiome associated with foods, few African labs are currently equipped to run these types of analyses. To address this need, a workshop was recently organized in Accra, Ghana to train participants in wet and dry lab approaches to food microbiome analysis. During the workshop *Analysis of the Microbiomes of Naturally Fermented Foods Training Course*, a modified DNA extraction method was applied to a wide range of foods and evaluated using amplicon sequence data. Bacterial populations of the samples were analysed by amplification of the V4 regions of the 16S rRNA gene and Illumina high-throughput sequencing. This study demonstrates that the modified method is suitable to analyse the bacterial populations of fermented foods produced from different raw materials by amplicon sequencing. The bacterial composition of different types of fermented foods produced throughout Africa was described and the structure of their bacterial populations was compared based on the raw material used and the conditions in which the fermentations were performed.

## Results and Discussion

### DNA extraction yield from fermented foods prepared from different raw materials

To test the performance of the DNA extraction method for analysis of the microbial diversity of different fermented foods, 40 spontaneously fermented African food samples with different characteristics (different raw material, production under different conditions or country of origin) were used (Table 1). The samples analysed in this study were produced in different regions of Africa, although they originate mainly from West African countries. Samples were grouped based on the raw material used as follows: cereal, cassava, dairy, locust beans and alcoholic beverages. DNA from samples S1 to S40 was extracted by the participants during the workshop, with 37 out of 40 samples yielding enough DNA to be analysed by amplicon-based metagenomics. Only three samples, one from the cassava group (S26), one from the alcoholic beverages group (S39) and one from the cereal group (S40) did not yield a measurable quantity of DNA (Table 1).

**Table 1:** List of samples used during the workshop and DNA extraction yield.

Sample code	Product name	Raw material	Fermentation group	Country	Production conditions	DNA concentration (ng/μl)
S1	Motoho	Sorghum	Cereal	South Africa	Laboratory	10.7
S2	Motoho	Sorghum	Cereal	South Africa	Laboratory	8.6
S3	Ogi	Sorghum	Cereal	Nigeria	Artisanal	502
S4	Ogi	Yellow maize	Cereal	Nigeria	Artisanal	434
S5	Mawe	Maize	Cereal	Benin	Commercial	224
S6	Mawe	Maize	Cereal	Benin	Commercial	195
S7	Mawe	Sorghum	Cereal	Benin	Commercial	48.6
S8	Mawe	Sorghum	Cereal	Benin	Commercial	145.4
S9	Boule d’akassa	Millet	Cereal	Burkina Faso	Artisanal	308
S10	Boule d’akassa	Millet	Cereal	Burkina Faso	Artisanal	318
S11	Gappal seche	Millet-milk	Dairy	Burkina Faso	Artisanal	147.8
S12	Nono	Milk	Dairy	Nigeria	Artisanal	64.6
S13	Wara	Milk	Dairy	Nigeria	Artisanal	145.4
S14	Fermented finger millet	Finger millet	Cereal	Kenya	Laboratory	264
S15	Fermented finger millet	Finger millet	Cereal	Kenya	Laboratory	244
S16	Obusera	Sorghum	Cereal	Uganda	Artisanal	39.8
S17	Tonton	Banana	Alcoholic	Uganda	Artisanal	188.4
S18	Millet dough	Millet	Cereal	Ghana	Artisanal	384
S19	Maize dough	Maize	Cereal	Ghana	Artisanal	250
S20	Cassava dough	Cassava	Cassava	Ghana	Artisanal	60.2
S21	Fura	Millet	Cereal	Ghana	Artisanal	284
S22	Millet dough	Millet	Cereal	Ghana	Laboratory	362
S23	Millet dough	Millet	Cereal	Ghana	Laboratory	354
S24	Kwerionik	Milk	Dairy	Uganda	Artisanal	13.1
S25	Ghee	Milk	Dairy	Uganda	Artisanal	44.6
S26	Gari	Cassava	Cassava	Ghana	Artisanal	UDL
S27	Kokonte	Cassava	Cassava	Ghana	Artisanal	135.8
S28	Dawadawa	Locust beans	Locust bean	Ghana	Artisanal	18.72
S29	Nunu	Milk	Dairy	Ghana	Artisanal	49.4
S30	Palm wine	Palm sap	Alcoholic	Nigeria	Artisanal	264
S31	Palm wine	Palm sap	Alcoholic	Nigeria	Artisanal	792
S32	Palm wine	Palm sap	Alcoholic	Nigeria	Artisanal	59.4
S33	Palm wine	Palm sap	Alcoholic	Nigeria	Artisanal	584
S34	Tej	Honey	Alcoholic	Ethiopia	Artisanal	240
S35	Teff dough	Teff	Cereal	Ethiopia	Artisanal	1040
S36	Teff dough	Teff	Cereal	Ethiopia	Artisanal	1480
S37	Dehulled maize dough	Dehulled maize	Cereal	Ghana	Commercial	23.8
S38	Dehulled maize dough	Dehulled maize	Cereal	Ghana	Commercial	21.6
S39	Burukutu	Sorghum	Alcoholic	Ghana	Commercial	UDL
S40	Pito	Sorghum	Cereal	Ghana	Commercial	UDL

The samples were obtained from rural areas (Artisanal), small scale producers (Commercial) or laboratory spontaneous fermentations (Laboratory). UDL = Under detection limit.

The mean and standard deviation of the DNA concentration from all the samples was 243.72 ± 300.93 ng/µl. Higher total DNA concentrations were obtained from cereal-based samples (289.23 ± 342.07 ng/µl) and alcoholic beverages (354.63 ± 275.7 ng/µl) compared to cassava (65.33 ± 68.04 ng/µl), dairy (77.49 ± 56.09 ng/µl) and locust bean (18.72 ng/µl) fermented samples.

### Bacterial diversity

Bacterial diversities of the 37 samples that yielded the required amount of DNA were analysed by 16S rRNA gene amplicon sequencing. DNA sequencing of the V4 amplicons by the Illumina MiSeq platform resulted in 3,321,898 paired-end sequence reads with an average of 89,781.03 ± 6,189.48 sequences per sample. Of these, 16.69% were discarded due to poor quality, reads not merging, or after being identified as chimeras; as a result of these steps, 2,767,337 high quality sequences were retained and analysed, with an average of 74,792.89 ± 5,069.18 sequences per sample. Distribution of reads per sample can be found in Supplementary Table S1.

**Figure 1:**
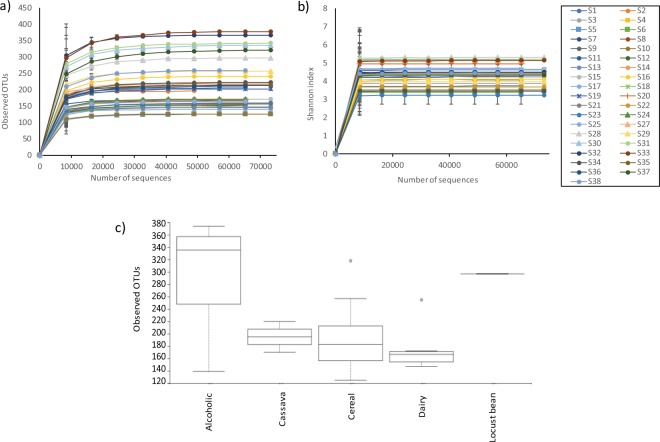
Rarefaction plots of (**a**) Observed OTUs and (**b**) Shannon index for each sample; (**c**) Boxplots of the observed OTUs per fermented sample for each fermentation group. Spots represent outliers.

Background DNA was removed by filtering out sequences assigned to chloroplast and mitochondrial taxonomic groups. Data were rarefied to 46,726 sequences per sample to avoid bias. As shown in Fig. 1(a,b), the rarefaction curves for the operational taxonomic units (OTUs) and Shannon index reach a plateau showing that the coverage was sufficient to capture the majority of the microbial diversity. Alpha diversity indexes (observed OTUs, Shannon, Faith’s phylogenetic diversity and Pielou’s evenness) were compared based on types of raw materials (cereal, cassava, dairy, locust beans and alcoholic beverages) and on the type of production process (artisanal, commercial or laboratory). Although alcoholic samples and the locust bean sample seemed to have a higher number of observed OTUs than cassava, cereal and dairy samples (Fig. 1c), no significant differences were found in any of the alpha diversity indexes compared.

**Figure 2:**
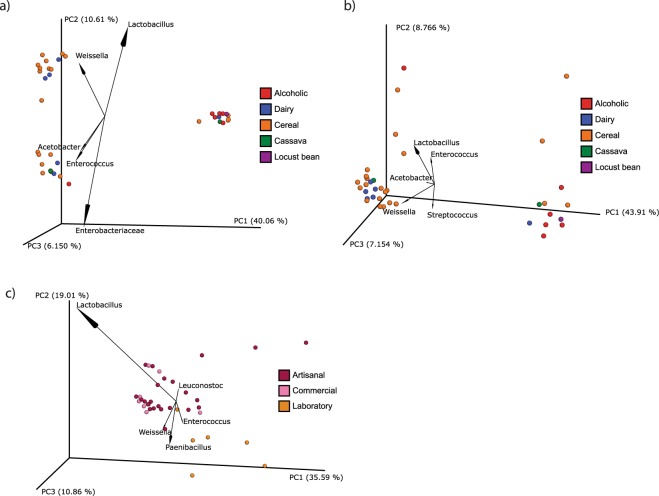
Principal coordinate analysis (PCoA) biplots showing (**a**) Jaccard, (**b**) unweighted UniFrac, (**c**) weighted UniFrac distances with samples coloured by raw material (**a,b**) or production conditions (**c**). The percentage of variation explained by the plotted principal coordinates is indicated in the axes. The arrows indicate the 5 taxonomic groups (at genera level) that contribute most to the indexes.

Principal coordinates analysis (PCoA) was performed to determine the effect of the raw material used and the production conditions on the microbial communities (Fig. 2). The differences based on the presence and absence of taxonomic groups were analysed using Jaccard (Fig. 2a) and unweighted UniFrac (Fig. 2b) distances. The fermentation samples grouped by the raw material correlated with the differences in the microbial populations observed when using the Jaccard (p-value 0.005) and unweighted UniFrac (p-value 0.005) distances. Most of the cereal and dairy samples clustered to one end of principal component 1 in the Jaccard (Fig. 2a) and unweighted UniFrac distances plots (Fig. 2b) (40.06% and 43.91% of variation explained, respectively), while most of the alcoholic samples cluster to the opposite end together with the locust bean sample. One of the cassava samples was grouped with the majority of the cereal and dairy samples, whilst the other was grouped with a large proportion of the alcoholic and locust bean samples. The differences between samples are mostly due to OTUs assigned to the *Lactobacillus, Weissella, Acetobacter* and *Enterococcus* genera and *Enterobacteriaceae* family for the Jaccard distance (vectors in Fig. 2a), and also for the unweighted UniFrac distance, but with the inclusion of the *Streptococcus* genera rather than the *Enterobacteriaceae* family (vectors in Fig. 2b). Cereal and dairy fermentations are both lactic acid fermentations, which likely explains why these samples share more OTUs than was observed with the alcoholic and locust bean fermentation samples.

**Figure 3:**
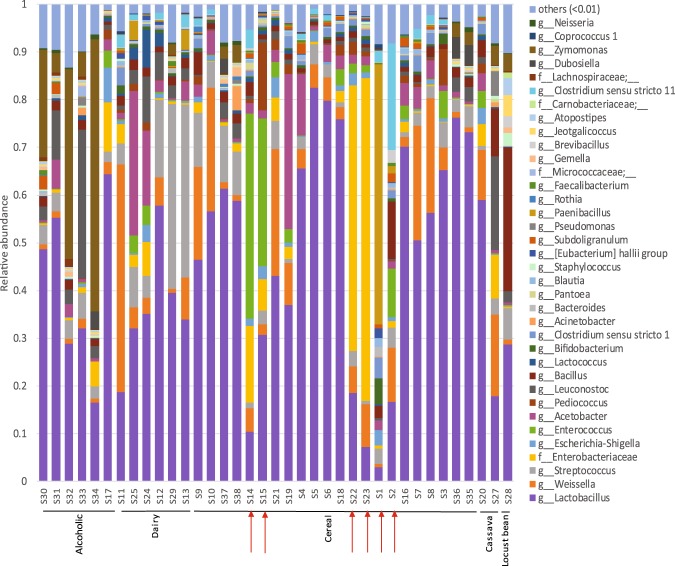
Relative abundance of the bacterial communities at the genus level. Taxonomic groups with abundance <1% were included in the group “others”. Where genera (g) could not be determined, family (f) level identification is shown. Arrows indicate samples produced under laboratory conditions.

In general, the most abundant bacteria in cereal and dairy fermentations were genera within the order *Lactobacillales* (phylum Firmicutes) such as *Lactobacillus, Weissella* and *Streptococcus*, except for samples produced under laboratory conditions (Fig. 3). Most of the OTUs within the *Lactobacillus* genus could not be classified at species level due to the short length of the 16S rRNA sequences. In general, most of the lactobacilli that could be classified at species level were identified as *Lactobacillus fermentum*. The relative abundance of the *Lactobacillus* species per sample can be found as Supplementary Fig. S2.

Other genera, such as *Acetobacter*, were found at relatively high abundances in some of the dairy and cereal fermented samples. These observations agree with most of the previous studies that describe the microbial populations of dairy^6^ and cereal^8,14,15^ foods produced in Africa. Bacterial genera that include potential pathogenic species such as *Escherichia* or *Clostridium* (cluster I and XI) were found in several cereal and dairy samples. It should be noted that the presence of the genus *Zymomonas* had not previously been described in fermented dairy or cereal samples. To our knowledge, this is the first time that this genus is reported in these types of food. In the sample of fermented cassava that showed a similar diversity to the cereal and dairy samples, *Lactobacillus* and *Weissella* were the most abundant genera, whereas the other sample showed high levels of bacteria from *Lactobacillus, Leuconostoc, Weissella, Bacillus* and *Enterobacteriaceae* (Fig. 3). Previous studies indicate that lactic acid bacteria from the *Lactobacillus, Weissella* and *Leuconostoc* genera^16,17^ together with the genus *Bacillus*^5,18^ were highly abundant in fermented cassava. However, the high abundance of *Zymomonas* observed in the latter sample was not reported in other studies. *Zymomonas movilis* has been previously used to produce ethanol from cassava waste^19^ but, to our knowledge, this is the first time that this genus is reported in non-alcoholic fermented cassava. In all the alcoholic samples, *Lactobacillus* and *Streptococcus* are among the most abundant genera. The genera *Zymomonas*, *Leuconostoc* and *Bacillus* are highly abundant in all the alcoholic samples except one, which clustered together with the lactic acid fermented samples. Previous studies described *Lactobacillus* and *Leuconostoc* as dominant groups in palm wine^20,21^. Although there is scarce information regarding the abundance of *Zymomonas* in these types of beverages, this genus has been typically isolated from alcoholic drinks^22^, such as palm wine and ripening honey^23^. In contrast to the previous samples, the locust bean undergoes an alkaline fermentation. Our results show that *Bacillus*, *Lactobacillus* and other genera within the *Lactobacillales* order are the predominant genera in this sample. A high abundance of *Bacillus* was expected in this sample, as this genus is responsible for the fermentation of the beans^24^. Although high levels of lactobacilli were not expected, they have previously been isolated from this type of product^25^. In this sample, several genera that have not been reported before in legume fermentations, such as *Zymomonas, Gemella, Brevibacillus, Jeotgalicoccus, Atopostipes* and members of the family *Carnobacteriaceae* were found. To compare the populations between the samples whilst considering the relative abundance of the taxa, weighted UniFrac distance was used (Fig. 2c). The production conditions explained the differences between the samples (p-value 0.002). Commercial and artisanal samples did not show significant differences between them (p-value 0.274), whilst samples produced in the laboratory were different from both artisanal (p-value 0.003) and commercial (p-value 0.002) samples. The differences between samples are largely due to OTUs within *Lactobacillus, Paenibacillus, Weissella, Enterococcus* and *Leuconostoc* genera (vectors in Fig. 2c). Analysis of Composition of Microbiomes (ANCOM) was used to predict significant differences in the relative abundance at the genera level. Based on the type of raw material used, significant differences (*W* = 281) were found in the *Lachnospiraceae* UCG-006 group. Alcoholic and locust bean samples showed a higher number of sequences (median = 52 and 61, respectively) than cereal, dairy and cassava samples (median = 1, 1 and 8, respectively). Nevertheless, this taxonomic group had very low abundance (less than 0.1%) in the samples in which it was present. Based on the production conditions, statistical differences (*W* = 113) were observed in the genus *Lactobacillus*. Artisanal and commercial samples showed a higher number of sequences (median of 33753 and 45292, respectively) assigned to *Lactobacillus* than those samples produced under laboratory conditions (median of 8429). This can be observed per individual sample in the relative abundance graph (Fig. 3), which also shows that most of the samples produced under laboratory conditions (red arrows, Fig. 3) have higher levels of bacteria from the *Enterobacteriaceae* family. In a recent study, higher levels of *Enterobacteriaceae* and *Pseudomonas* were observed in fermentations performed in the laboratory compared to household fermentations^8^. The lower relative abundance of lactobacilli in the fermented samples produced under laboratory conditions could be explained by the use of a sterile environment and sterilized tools. The environmental microbiota has been described as playing an important role in the fermentation dynamics^26^, and tools used during some fermentations have been pointed to as a source of lactobacilli^7,27^. This should be considered when performing fermentation experiments under laboratory conditions, as spontaneous fermentations may not develop equally in the laboratory and in-field production.

## Conclusion

Most of the previous reports on the bacterial diversity of African fermented foods were based on culture-dependent analysis. Nevertheless, these methods present several limitations, which are resolved by culture-independent methods. In this study, a modified method to extract DNA from different food matrices is proposed. The resultant DNA was used to analyse the bacterial diversity of traditional African fermented products by 16S rRNA gene amplicon sequencing. In this study, the genus *Zymomonas* has been reported for the first time in dairy, cereal, cassava and locust bean fermentations. This shows the importance of using culture-independent methods to study the bacterial communities of African fermented food. Results show that the genus *Lactobacillus* is less abundant in fermentations performed in laboratory conditions compared to artisanal or commercial fermentations. This is an important finding as this could affect the interpretation of results observed in model fermentations.

## Materials and Methods

### Sample collection and storage

In this study, 40 food samples were collected and used by the participants of the Workshop “Analysis of the Microbiomes of Naturally Fermented Foods”, held from the 5^th^ to the 9^th^ of February 2018 in Accra (Ghana). The fermented samples, produced from 10 different raw materials (sorghum, maize, millet, banana, cassava, locust beans, palm tree sap, teff, honey and milk), were collected from 8 different African countries (Ghana, Nigeria, Benin, Burkina Faso, Uganda, Kenya, Ethiopia and South Africa). They were obtained from rural areas (artisanal), small-scale producers (commercial) or laboratory spontaneous fermentations (laboratory) (Table 1) and kept at -20 °C until the DNA was extracted. Samples were grouped according to the type of raw material: cereals, cassava, dairy, locust beans and alcoholic beverages (Table 1).

### Pre-processing, DNA extraction and DNA quantification

To extract DNA from fermented samples, the FastDNA Spin Kit for Soil (MP Biomedicals, UK) was used in conjunction with a pre-processing step which separates the microbial cells from large solid particles present in the sample. See Supplementary methods for a description of the optimization of the method. First, 20 g of the sample were mixed with 10 ml of cold ultrapure H_2_O by vigorous vortexing. The solid particles were removed by centrifugation at 800 × *g* for 1 min at 4 °C and the supernatant was retained. A further 10 ml H_2_O was added and the process was repeated three times in total; a final volume of approximately 30 ml supernatant was obtained. Cells were harvested from the particle-free supernatants by centrifugation at 3000 × *g* for 20 min at 4 °C. The supernatant was discarded and the pellet was washed three times using 1 ml PBS buffer. After centrifugation at 14,000 × *g* for 2 min, the pellet was resuspended in 978 µl sodium phosphate buffer and 122 µl MT buffer and incubated for 1 h at 4 °C and homogenized for 60 s at a speed setting of 6.5 m/s, using a FastPrep-24 instrument (MP Biomedicals, UK). This process was repeated three times and samples were kept on ice for 5 min between each homogenization step. Otherwise, DNA extraction was performed according to the manufacturer’s instructions. The extracted DNA was resuspended in 50 μl elution buffer.

Total DNA extracted from the fermented samples was quantified fluorometrically by a Qubit 3.0 fluorometer (Invitrogen, Carlsbad, CA) using the Qubit dsDNA BR Assay Kit (Invitrogen), or the Qubit dsDNA HS Assay Kit (Invitrogen) when the concentration of DNA was <10 ng/µl.

### Illumina high-throughput sequencing

16S rRNA gene PCR amplification and sequencing was performed by Novogene (HK) Company Limited (Hong Kong). The V4 hypervariable region of the 16S rRNA gene was amplified using specific primers 515 F and 806R^28^. All PCR reactions were carried out with Phusion® High-Fidelity PCR Master Mix (New England Biolabs, USA). The libraries generated with NEBNext Ultra II DNA Library Prep Kit for Illumina (New England Biolabs, England) were sequenced using paired-end Illumina sequencing (2 × 250 bp) on the HiSeq. 2500 platform (Illumina, USA).

### Sequence analysis

Sequencing data, for the 37 samples that yielded a perceptible concentration of DNA (>0.2 ng/μl), were analysed using the Quantitative Insights Into Microbial Ecology 2 (QIIME2) 2018.8 software^29^. The demultiplexed paired-end reads were filtered of substitution and chimera errors and merged using DADA2^30^. Bacterial taxonomic assignment was performed at 97% similarity using a Naive Bayes classifier trained on the Silva version 132 99% OTU database^31^, where the sequences have been trimmed to only include 250 bases from the V4 region bound by the 515 F/806 R primer pair. Alpha diversity was analysed using observed OTUs, Shannon, Pielou’s evenness, and Faith’s phylogenetic diversity indexes. Rarefaction curves were computed using OTUs and Shannon index. Jaccard, unweighted and weighted UniFrac distances were used to generate beta diversity PCoA biplots, which were visualised using the Emperor tool^32^.

### Statistical analysis

Wilcoxon sign test (2-tailed) was used to compare the DNA yielded by the two extraction methods tested using IBM SPSS Statistics Version 22.0. software (IBM Corp., USA). Significant differences in alpha diversity between groups were calculated using the alpha-group-significance script in QIIME2, which performs the Kruskal-Wallis test. Differences in beta diversity between groups were analysed using PERMANOVA including pairwise test (Anderson, 2001). Significant differences in the bacterial community structure amongst the groups were evaluated by Analysis of Composition of Microbiomes (ANCOM)^33^. A p-value ≤ 0.05 was considered statistically significant.

## Supplementary information

SUPPLEMENTARY INFORMATION

## Data Availability

The datasets generated and analysed during the current study are available in the SRA database under the accession number PRJNA532858.
