# Supplementary material for: Comparison of the microbial composition of African fermented foods using amplicon sequencing
Source: Sci Rep. 2019 Sep 25;9:13863. doi: 10.1038/s41598-019-50190-4 (PMC6761159; doi:10.1038/s41598-019-50190-4)

SUPPLEMENTARY INFORMATION

Comparison of the microbial composition of African fermented foods using amplicon sequencing

Maria Diaz[1]*, Lee Kellingray[2]*, Nwanneka Akinyemi[3], Oyetayo Adefiranye[3†], Arinola B Olaonipekun[4]†, Geoffroy Romaric Bayili[5]†, Jekwu Ibezim[3]†, Adri du Plessis[4]†, Marcel Houngbédji[6]†, Deus Kamya[7]†, Ivan Muzira Mukisa[7]†, Guesh Mulaw[8]†, Samuel Manthi Josiah[9]†, William Onyango Chienjo[10], Amy Atter[11]†, Evans Agbemafle[11]†, Theophilus Annan[11]†, Nina Bernice Ackah[11]†, Elna Buys[4], D. Joseph Hounhouigan[6], Charles Muyanja[7], Jesca Nakavuma[7], Damaris Achieng Odeny[9], Hagretou Sawadogo-Lingani[5], Anteneh Tesfaye Tefera[12], Wisdom Amoa-Awua[11], Mary Obodai[11], Melinda J Mayer[2], Folarin A. Oguntoyinbo[3,13], Arjan Narbad[2]

[1]Food Innovation and Health Institute Strategic Programme, Quadram Institute Bioscience, Norwich Research Park, Norwich, United Kingdom, [2]Gut Microbes and Health Institute Strategic Programme, Quadram Institute Bioscience, Norwich Research Park, Norwich, United Kingdom, [3]Department of Microbiology, Faculty of Science, University of Lagos, Lagos, Nigeria, [4]Consumer and Food Science Department, University of Pretoria, Pretoria, South-Africa, [5]Département Technologie Alimentaire DTA/IRSAT/CNRST, Ouagadougou, Burkina Faso, [6]Laboratoire de Sciences des Aliments, Université d'Abomey-Calavi, Benin, [7]Department of Food Technology & Nutrition, Makerere University, Kampala, Uganda, [8]Department of Microbial, Cellular and Molecular Biology, Addis Ababa University, Addis Ababa, Ethiopia,

[9]International Crops Research Institute for Semi-arid Tropics (ICRISAT), Nairobi, Kenya, [10]Department of Food Science and Technology, The Technical University if Kenya, Kenya, [11]CSIR-Food Research Institute, Accra, Ghana, [12] Institute of Biotechnology, Addis Ababa University, Ethiopia, [13]A.R. Smith Department of Chemistry and Fermentation Sciences, Appalachian State University, Boone, North Carolina.

† Authors contributed equally to this manuscript

* Co-corresponding authorship

#Address correspondence to M. Diaz (Maria.Diaz@quadram.ac.uk) and Lee Kellingray (Lee.Kellingray@quadram.ac.uk).

Supplementary Table S1. Number of reads per sample after each filtering step.

Sample code	Sequence count	filtered	denoised	merged	non-chimeric	non-background DNA
**S1**	80091	72462	72462	71119	68837	68748
**S2**	84950	78859	78859	75296	60689	50148
**S3**	93400	87568	87568	84947	73746	73473
**S4**	84943	79578	79578	77385	67803	67649
**S5**	95390	89632	89632	87139	77171	76528
**S6**	90807	85735	85735	83411	72911	72451
**S7**	98320	92551	92551	89414	77812	75867
**S8**	92091	86995	86995	84235	72999	71192
**S9**	95647	88130	88130	84908	73625	72575
**S10**	96998	91385	91385	88836	77144	76962
**S11**	99396	91135	91135	88514	80812	46726
**S12**	86183	80750	80750	78297	70151	69974
**S13**	98058	91233	91233	88430	78064	77855
**S14**	97558	90451	90451	87434	82066	81789
**S15**	92134	86234	86234	83760	75757	75585
**S16**	98029	91910	91910	89317	82595	82576
**S17**	88221	82590	82590	80028	73782	73764
**S18**	86346	81540	81540	79446	72808	72803
**S19**	87116	81919	81919	79728	75074	75054
**S20**	80342	75610	75610	73272	67397	67362
**S21**	92292	86936	86936	84406	77573	77569
**S22**	90615	83988	83988	82171	78836	78825
**S23**	86554	80557	80557	78977	74448	74249
**S24**	81200	76069	76069	73354	68590	68590
**S25**	80686	75272	75272	73095	67793	67790
**S27**	80979	76837	76837	74254	69855	68032
**S28**	97441	92379	92379	88465	80147	80080
**S29**	89653	84386	84386	81697	75908	75859
**S30**	88070	83893	83893	81143	75354	75312
**S31**	99925	95544	95544	92248	86427	86387
**S32**	84162	80323	80323	78495	75257	75194
**S33**	89739	85444	85444	82499	77529	77402
**S34**	82868	79047	79047	77380	74696	74379
**S35**	93484	87636	87636	85001	78887	78257
**S36**	86226	82264	82264	79919	73367	72013
**S37**	91450	86776	86776	84202	80161	79629
**S38**	80534	76494	76494	74267	71266	70931

Supplementary Figure S1. Comparison of DNA yielded by extraction methods M1 (white bars) and M2 (black bars). a) Mean and standard deviation of the total DNA concentration (ng/µl) extracted from samples O1 to O4; b) Mean and standard deviation of the ng of bacterial DNA per ng of total DNA extracted per sample.

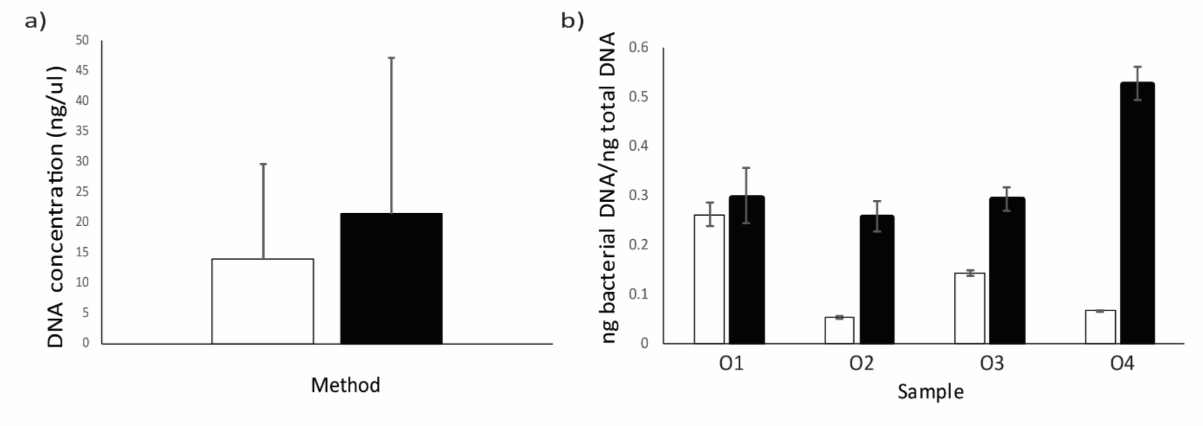

Supplementary Methods: Optimization of bacterial DNA extraction yield

In our previous attempts to extract DNA from fermented cereals, very low yields were observed (data not shown). In this study, the FastDNA SPIN Kit for soil, which uses mechanical lysis to break the cells, was used and the effect of a pre-treatment of the sample on the yield of the extraction was tested. DNA was extracted from four cereal-based fermented samples (Supplementary Table S1) using methods M1 (DNA extraction without pre-treatment) and M2 (DNA extraction with pre-treatment).

Supplementary Table S2. List of samples used to compare the DNA extraction yield of methods M1 and M2.

**Sample code**	**Product name**	**Raw material**	**Country**	**Production conditions**
**O1**	Maasa	Millet	Ghana	Artisanal
**O2**	Maasa	Millet	Ghana	Artisanal
**O3**	Kenkey	Maize	Ghana	Artisanal
**O4**	Maasa dough	Millet	Ghana	Artisanal

Total DNA extracted from the fermented samples was quantified fluorometrically by a Qubit 3.0 fluorometer (Invitrogen, Carlsbad, CA) using the Qubit dsDNA BR Assay Kit (Invitrogen), or the Qubit dsDNA HS Assay Kit (Invitrogen) when the concentration of DNA was <10 ng/µl. Bacterial DNA was quantified by quantitative PCR (qPCR) of the 16S rRNA gene using a SensiFAST SYBR No-ROX Kit (Bioline, UK) and a ViiA 7 Real-Time PCR System (Applied Biosystems, USA). The reaction consisted of 2x SensiFAST SYBR No-ROX Mix, 0.4 µM primers 515F and 806R (Caporaso et al., 2011) and 0.1 ng DNA as template. A calibration curve ($R^2 > 0.99$) for calculation of bacterial DNA quantity was generated based on gDNA extracted from *Lactobacillus plantarum* FI11116 (isolated from *ogi*, a traditional fermented maize, unpublished).

Although no statistical differences were found in the total DNA yielded by both methods, differences were observed in some samples. Method M2 yielded more total DNA in sample O4 than method M1 (58 vs 1.28 ng/µl), and similar yields in samples O2 (1.81 ng/µl vs 1.01 ng/µl) and O3 (21.2 vs 19.4). Higher DNA yield was obtained with method M1 for sample O1 (33.8 vs 4.04 ng/µl). To check whether the differences between the amount of total DNA extracted with both methods was due to a change in the amount of bacterial DNA extracted or if it was background DNA (DNA from the raw materials), qPCR of the bacterial 16S rRNA gene was performed. A statistically significant increase (p-value= 0.011) was observed in the bacterial DNA extraction yield of method M2 compared to method M1. As shown in Fig. 1b, method M2 yielded more bacterial DNA per ng of total DNA than method M1 (7.8-fold increase for sample O4, 4.75-fold increase for sample O2, 2.05-fold for sample O3 and 1.12-fold increase for sample O1). These results show that method M2 reduces the background DNA from the food matrices.

Method M1 could overestimate the amount of bacterial DNA used when applying high-throughput techniques.

Supplementary Figure S2. Relative abundance of bacterial community at species level for OTUs within the genus *Lactobacillus.*

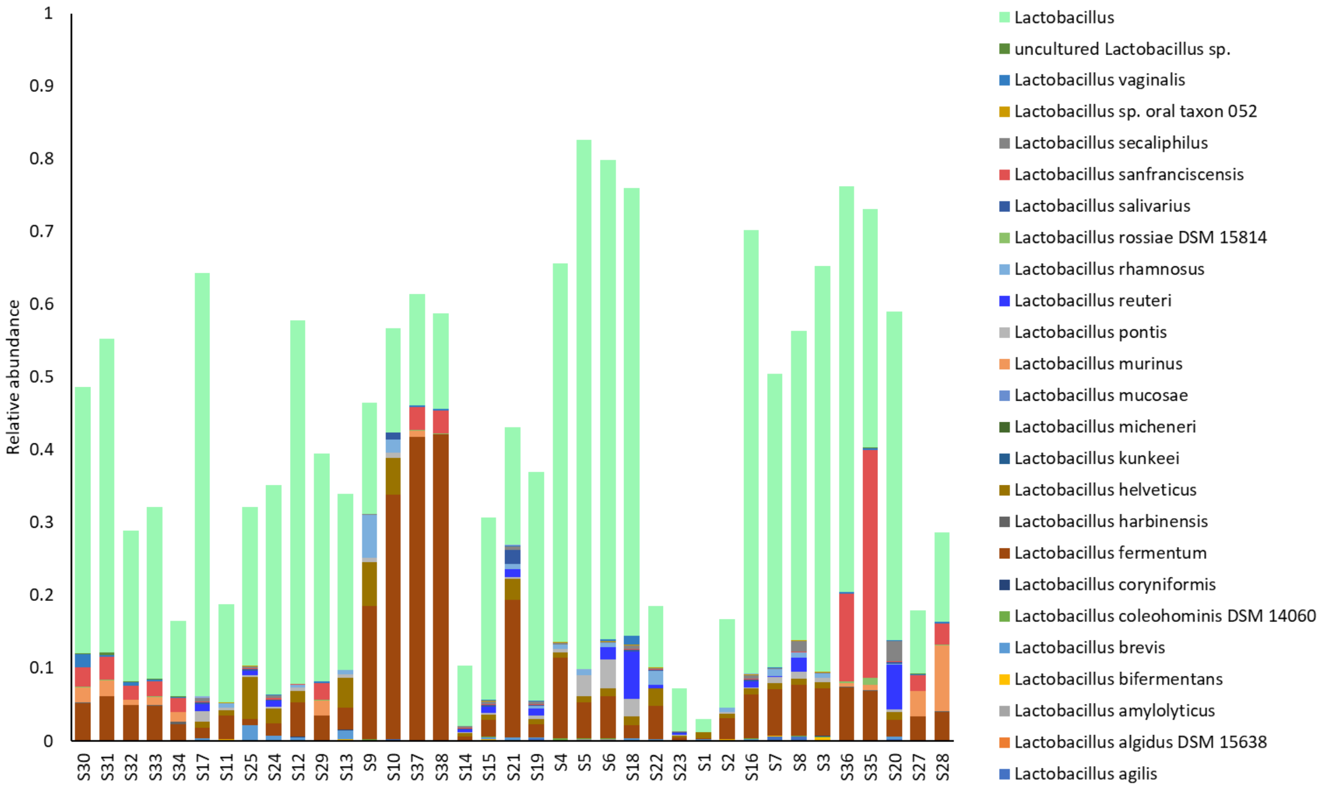

Supplement: Supplementary file 1 — SUPPLEMENTARY INFORMATION [file 41598_2019_50190_MOESM1_ESM.pdf]
